# The Siamese White Elephants

**Published:** 1882-02

**Authors:** 


### THE SIAMESE WHITE ELEPHANTS—A LEAF FROM PROFESSOR WARD'S NOTE-BOOK.

"Of course I did not leave the king's palace without going to see his famous white elephants, of which he now possesses five. They are in a long block of buildings at the rear of the arsenal. Here each elephant has an entire, distinct stable appointed to its use, with the sleeping apartments of its score of grooms and feeders in one end. The stable is a large high hall, with plain floor and walls, and at one end a small seated Buddha image with little lamps burning in front. The great beast stands on a handsomely built pedestal, raised about a foot from the floor, with its top just large enough to hold him. On one side of this, near the front and the rear, are two high, stout posts, gilded and otherwise gaudily ornate. To these the royal captive is chained with velvet-covered ropes by one fore and one hind leg. He stands proudly yet restlessly on his contracted throne, and lashes his trunk and sways his heavy head and tusks around in an imperious, lordly manner, trumpeting now and then until the whole hall trembles with the deafening reverberation. His keepers are very attentive, and constantly deal out to him dainty mouthfuls of bananas, pineapples, short sticks of sugar-cane, and little bundles of sweet, fresh grass from a huge pile of these which is heaped near by. Every one of his wants is assiduously attended to; when he is seen to itch in any part of his body his royal hide is promptly scratched with a small iron rake-like instrument with a long handle; his eyes are reverently wiped, and he has a cool sponge bath every hour or two of day and night during the hot season. Over the door of each stable is a large, richly ornamented sign of gold letters, giving the inmate's full regal name and title. This the guide reads to you in reverently hushed breath; indeed, these names, like that of the king, it is not proper for any Siamese to speak aloud.

"Nothing can equal the veneration of this people for the white elephant; the king must have at least one as a palladium for his own life and the prosperity of the empire. The real cause of this reverence for the great albino pachyderm is that he is supposed to be the incarnation either of some past or of some future Buddha, and will, therefore, bring blessings on the country which possesses and cherishes so great a treasure. Time was when these beasts were duly worshiped by king and people; their stables were palaces; they were fed from golden dishes, and wore heavy gold rings upon their tusks and were fettered with golden chains. Even now the populace fall with their heads to the ground as they are led out richly caparisoned on state occasions, while the royal officers and even the king himself always make them obeisance in passing. The finder of a white elephant is loaded with honors and emoluments, taking his place at one step among the nobles of the kingdom. They come from the Laos territory far to the north, and are brought down the river on rafts magnificently decked out, and accompanied by guards of honor and bands of music. At Aguthia, sixty miles up the river, they are met by the king with his entire royal retinue, who in the grandly adorned state barges follow the noble beast to its future home, with great parade and rejoicing.

"It is a late moment for me to state that these 'white' elephants have not at all the fulness or the depth of that color which we usually understand, or which the adjective demands. Over most of their body they have the dull, muddy lead-color common to this beast. Only in spots upon their legs and in patches on shoulders, neck, sides of head and inside of ears the skin is of a dirty cream color, not quite pleasant to look at, besides being abundantly splashed with coarse freckles. No one would think of calling them white. One only of their number, the fifth and last one obtained, is of a faint brick red over his entire body, which gives him an odd and not altogether unpleasant appearance. He is, moreover, young, lively and good-natured, and salaams by raising his trunk straight and high above his head to all well-dressed visitors in a way which quite scandalizes his keepers, who have taught all the others to reserve that salute solely for the king. Were he not himself too royal to be whipped, I dare say that this merry pachyderm might soon be taught to recognize the honor reserved to royalists. White animals of all kinds—albino accidents—seem to be rather frequent in Siam, and the people of all grades have a sort of religious feeling of respect toward them. White monkeys and white magpies are kept by the nobles in rich, costly cages. The bonzes or priests salute a white cock which they may meet in their walks, when they would not salute a prince on like occasion."

---

It was the quaint saying of a dying man, who exclaimed: "I have no fear of going home. God's finger is on the latch, and I am ready for him to open the door. It is but the entrance of my father's house."

